# Bioreactor Platform for Biomimetic Culture and **in situ** Monitoring of the Mechanical Response of **in vitro** Engineered Models of Cardiac Tissue

**DOI:** 10.3389/fbioe.2020.00733

**Published:** 2020-07-14

**Authors:** Diana Massai, Giuseppe Pisani, Giuseppe Isu, Andres Rodriguez Ruiz, Giulia Cerino, Renato Galluzzi, Alessia Pisanu, Andrea Tonoli, Cristina Bignardi, Alberto L. Audenino, Anna Marsano, Umberto Morbiducci

**Affiliations:** ^1^PolitoBIOMed Lab, Department of Mechanical and Aerospace Engineering, Politecnico di Torino, Turin, Italy; ^2^Interuniversity Center for the Promotion of the 3Rs Principles in Teaching and Research, Turin, Italy; ^3^Department of Surgery, University Hospital of Basel, Basel, Switzerland; ^4^Department of Biomedicine, University of Basel, Basel, Switzerland; ^5^Department of Mechanical and Aerospace Engineering, Politecnico di Torino, Turin, Italy

**Keywords:** bioreactor, mechanical stimulation, *in situ* monitoring, engineered cardiac tissues, cardiac tissue maturation, cardiac tissue engineering

## Abstract

In the past two decades, relevant advances have been made in the generation of engineered cardiac constructs to be used as functional *in vitro* models for cardiac research or drug testing, and with the ultimate but still challenging goal of repairing the damaged myocardium. To support cardiac tissue generation and maturation *in vitro*, the application of biomimetic physical stimuli within dedicated bioreactors is crucial. In particular, cardiac-like mechanical stimulation has been demonstrated to promote development and maturation of cardiac tissue models. Here, we developed an automated bioreactor platform for tunable cyclic stretch and *in situ* monitoring of the mechanical response of *in vitro* engineered cardiac tissues. To demonstrate the bioreactor platform performance and to investigate the effects of cyclic stretch on construct maturation and contractility, we developed 3D annular cardiac tissue models based on neonatal rat cardiac cells embedded in fibrin hydrogel. The constructs were statically pre-cultured for 5 days and then exposed to 4 days of uniaxial cyclic stretch (sinusoidal waveform, 10% strain, 1 Hz) within the bioreactor. Explanatory biological tests showed that cyclic stretch promoted cardiomyocyte alignment, maintenance, and maturation, with enhanced expression of typical mature cardiac markers compared to static controls. Moreover, *in situ* monitoring showed increasing passive force of the constructs along the dynamic culture. Finally, only the stretched constructs were responsive to external electrical pacing with synchronous and regular contractile activity, further confirming that cyclic stretching was instrumental for their functional maturation. This study shows that the proposed bioreactor platform is a reliable device for cyclic stretch culture and *in situ* monitoring of the passive mechanical response of the cultured constructs. The innovative feature of acquiring passive force measurements *in situ* and along the culture allows monitoring the construct maturation trend without interrupting the culture, making the proposed device a powerful tool for *in vitro* investigation and ultimately production of functional engineered cardiac constructs.

## Introduction

The native myocardium is a complex tissue composed of an anisotropic network of elongated, tightly interconnected cardiomyocytes (CMs) and cardiac fibroblasts (FBs) embedded in a collagen-rich extracellular matrix (ECM) with a dense supporting vasculature. Defined pacemaker cells and electrically coupled CMs are responsible for the coordinated and cyclic wringing contraction of the heart. Myocardial infarction can cause irreversible damage to the myocardium; indeed, its inability to regenerate together with consequent cardiac fibrosis and irreversible ventricular remodeling can significantly impair heart function over time (Murtha et al., 2017). In the past two decades, relevant advances have been made in the development of three-dimensional (3D) engineered cardiac tissues (ECTs) aimed to recapitulate the structural and functional properties of the native myocardium (Eschenhagen and Zimmermann, 2005; Ye et al., 2013; Zimmermann, 2013; Hirt et al., 2014b; Ogle et al., 2016; Fujita and Zimmermann, 2017; Weinberger et al., 2017). Although these approaches still did not attain complete functionality and immunosafety suitable for clinical translation, ECTs are widely used as *in vitro* models for basic cardiac research (Engelmayr et al., 2008; Tiburcy et al., 2011), for drug screening (El-Armouche et al., 2007; Hansen et al., 2010; Schaaf et al., 2011; Vunjak-Novakovic et al., 2014; Mannhardt et al., 2016; Marsano et al., 2016), and for investigating cardiac disease (de Lange et al., 2011; Hirt et al., 2012; Occhetta et al., 2018; Chen and Vunjak-Novakovic, 2019; Mytsyk et al., 2019), with the ultimate but still challenging goal to act as functional substitutes for replacement of damaged myocardium (Zimmermann and Eschenhagen, 2003; Zimmermann et al., 2006; Lesman et al., 2010; Menasché et al., 2015; Riegler et al., 2015; Weinberger et al., 2016; Miyagawa et al., 2017; Tiburcy et al., 2017; Frati et al., 2019; Belviso et al., 2020).

Successful strategies for the *in vitro* generation of functional ECTs require a synergistic combination of appropriate cells, scaffolds, and cardiac-like biochemical and biophysical signals (Bilodeau and Mantovani, 2006; Vunjak-Novakovic et al., 2010). In particular, it has been demonstrated that providing biomimetic mechanical stimulation within dedicated bioreactors promotes growth, maturation, and contractile function of ECTs together with the development of a myocardium-like anisotropic architecture (Massai et al., 2013; Liaw and Zimmermann, 2016; Parsa et al., 2016; Stoppel et al., 2016). In several studies, phasic mechanical stretch (isotonic load) was delivered to ECTs for mimicking the cyclic diastolic filling of the ventricles, leading to morphological and phenotypic changes, referred to as hypertrophy, accompanied by improved cell proliferation, organized matrix formation, and enhanced contractile performance (Fink et al., 2000; Akhyari et al., 2002; Zimmermann et al., 2002; Naito et al., 2006; Birla et al., 2007; Tulloch et al., 2011; Salazar et al., 2015). However, due to the absence of *in situ* monitoring, in these approaches ECTs were transferred to dedicated measurement devices to characterize their mechanical response. This made force measurements mainly used as end-point analyses, without a continuous characterization of the stage-specific maturation of the constructs. In parallel works, ECTs were suspended between load-adjustable mounts or flexible posts for mimicking the native auxotonic contractions against the hydrostatic pressure of the circulation (Zimmermann, 2013; Hirt et al., 2014a; Liaw and Zimmermann, 2016). Under auxotonic load, ECTs progressively induced the deflection of the posts by gradually adapting and increasing their contraction force, with consequent structural and functional tissue maturation (Zimmermann et al., 2006; Hansen et al., 2010; Schaaf et al., 2011; Boudou et al., 2012; Mannhardt et al., 2016; Tiburcy et al., 2017). This approach enabled culturing miniaturized ECTs in modular platforms designed for simultaneous individual control of culture environment and for automatic video-optical evaluation of contractile activity, crucial features in the perspective of automated high-throughput analyses for drug screening (Hansen et al., 2010; Schaaf et al., 2011; Mannhardt et al., 2016). Despite these advantages, auxotonic load requires long culture periods for tissue adaptation. Furthermore, flexible posts do not allow to either control the mechanical load imposed to the ECTs or adapt the load to the construct maturation stage, so that muscle training or pathological stimulation regimes cannot be applied (Liaw and Zimmermann, 2016).

In order to overcome these limitations and being inspired by the challenge to adapt the mechanical conditioning to the actual maturation stage of the cultured constructs, we developed an automated bioreactor platform for tunable cyclic stretch culture and *in situ* non-destructive monitoring of the passive mechanical response of the cultured ECTs (Pisani, 2016). To demonstrate the bioreactor platform performance and to investigate the impact that cyclic stretch has on maturation and contractility of engineered models of cardiac tissue, we produced 3D annular constructs based on neonatal rat CMs and FBs embedded in fibrin hydrogel (Bian et al., 2009; Black et al., 2009; Hansen et al., 2010). The hydrogel technique was adopted since it allows to define the ECT shape by using customized casting molds and to support cell spreading and connections throughout the constructs (Hirt et al., 2014b). Fibrin hydrogel was selected since it is easy to pour, tunable in stiffness, and FDA-approved (Jockenhoevel et al., 2001). Fibrin-based ECTs were subjected to controlled cyclic stretch within the proposed bioreactor platform, and their mechanical response was *in situ* monitored along the culture. The effect of cyclic stretch conditioning was analyzed in terms of cell organization, cardiac marker expression, collagen content, mechanical properties, and electrical functionality.

## Materials and Methods

### Bioreactor Platform Design and Working Principle

#### Design Requirements

Specific requirements guided the design phase. The bioreactor platform should provide tunable uniaxial cyclic stretch in the range of physiological and pathological stimuli experienced by human cardiac tissue *in vivo* (strain = 5–100%, frequency = 1–4 Hz; Kuznetsova et al., 2008). Furthermore, the device should enable *in situ* monitoring of the passive mechanical response of the cultured constructs, using non-destructive methods. Modularity and customization requirements came from the need to guarantee versatility of the device, intended to be used with multiple and different constructs and for several cell/tissue applications. For Good Laboratory Practice (GLP) compliance, the bioreactor platform should be easy to use and to clean with standard tools and techniques commonly available in a cell biology laboratory, and the culture chamber components should be cytocompatible and autoclavable. In addition, the bioreactor platform should have an overall size suitable to be assembled under laminar flow hood and placed on a standard incubator shelf (40 cm × 30 cm × 40 cm).

#### Design, Components, and Working Principle

**FIGURE 1:**
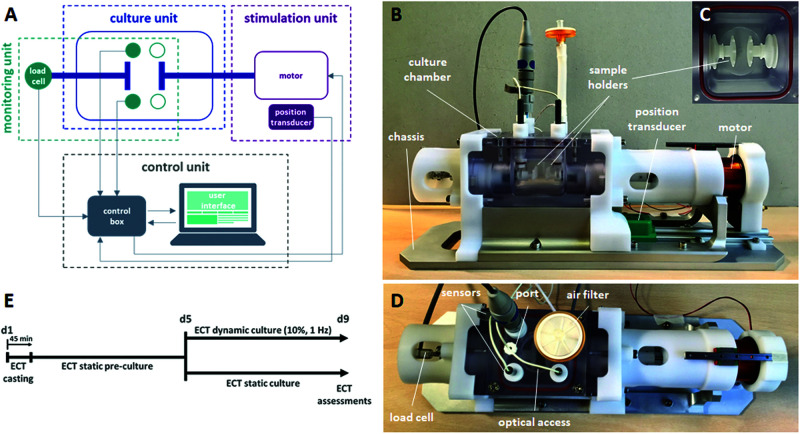
Bioreactor platform and experimental plan. **(A)** Scheme of the connections among the bioreactor units: culture, stimulation, monitoring, and control units. **(B)** Lateral view of the bioreactor platform showing the chassis, the culture chamber, the sample holders, the position transducer, and the motor. **(C)** Top view of the sample holders within the culture chamber. **(D)** Top view of the bioreactor platform showing the load cell and, on the culture chamber lid, the sensors, the medium sampling/replacement port, the air filter, and the optical access. **(E)** Experimental plan of the ECT culture, showing the static pre-culture time up to day 5 (d5) and the following culture in either dynamic or static condition up to day 9 (d9).

In order to accomplish all the abovementioned requirements, the proposed bioreactor platform consists of (1) a culture unit, for housing the constructs; (2) a monitoring unit, for *in situ* monitoring of culture environment and construct mechanical response; (3) a stimulation unit, for providing tunable uniaxial cyclic stretch; and (4) a control unit, for controlling the stimulation, acquiring and recording the sensor signals, and guaranteeing communications from and to the user (Figure 1A). In detail, the culture unit is composed of a polycarbonate culture chamber (140 mm × 80 mm × 75 mm with a priming volume of ∼100 ml, Figure 1B), in which interchangeable polyoxymethylene (POM) sample holders, designed for ring- or patch-shaped constructs (Supplementary Figure 1), can be mounted (Figure 1C). One sample holder is coupled to an AISI 316L stainless-steel through-shaft, externally connected to the motor, while the other sample holder is fixed and mounted on an AISI 316L stainless-steel through-shaft, externally connected to a load cell. This setup allows connection of the ECTs to the motor on one side and the force sensor on the other, while silicone bellows (J-Flex rubber, United Kingdom) fitted on the internal side of both through-shafts assure watertightness and sterility maintenance of the culture chamber. The polycarbonate lid, screwed on the vessel and sealed by a silicone O-ring, can accommodate up to four immersed sensors and is equipped with one medium sampling/replacement port and one air filter. A central optical access allows for the visual inspection of the constructs during culture (Figure 1D). The monitoring unit can include pH (InLab Ultra-Micro sensor, Mettler Toledo, Switzerland) and dissolved oxygen (MI-730 Micro-Oxygen Electrode, Microelectrodes Inc., United States) sensors inserted in the lid and a load cell with a 10-N full scale (XFTC300, Measurement Specialties TE Connectivity, United States) connected to the fixed holder through-shaft for *in situ* non-destructive monitoring of the mechanical response of the constructs (Figure 1D). The stimulation unit is based on a linear voice coil motor (GVCM-051-064-01, Moticont, United States), which is connected by a through-shaft to one sample holder and provides sinusoidal uniaxial cyclic stretch with strain values in the range of 5–100% for constructs of 2–15-mm length, with a stimulation frequency range of 1–6 Hz. A contactless linear position transducer (ONP1-A, Gefran, IT) is located under the motor to measure its displacement. An aluminum/POM chassis houses the culture chamber, the monitoring unit, and the stimulation unit (Figure 1B) to be placed within a standard cell culture incubator (37°C, 5% CO_2_, and 90% humidity). The control unit is composed of a laptop, with a purpose-built software, and hardware components contained in a customized control box. In detail, the CompactRIO-9075 real-time controller (National Instruments, United States) was selected to drive the monitoring and control of the bioreactor. The motor is controlled by a feedback-loop strategy based on the position transducer and a drive motion module (NI 9505 Full H-Bridge, National Instruments, United States). A numerical lumped-element model (MATLAB & Simulink, MathWorks, United States) of the control loop of the motor was developed and supported the tuning of the stimulation unit control (see details in Supplementary Material). A signal conditioner (ARD154, Measurement Specialties TE Connectivity, United States) amplifies the signal of the load cell and increases measurement resolution, improving the signal-to-noise ratio. The analog signals collected from the position transducer, the sensors, and the load cell are processed and digitalized by the NI 9219 acquisition module (National Instruments, United States). Finally, a user-friendly software interface, developed using the LabView^®^ graphical system design platform (National Instruments, United States), allows the user to set the mechanical stimulation parameters (displacement mode, displacement value, frequency), to be guided during the sensor calibration protocols, and to monitor and save the data collected from the monitoring unit (Supplementary Figure 2). The bioreactor components were designed using the commercial computer-aided design (CAD) software SolidWorks (Dassault Systemes, France). Working prototypes of the sample holders were 3D printed (Stratasys uPrint SE Plus, Stratasys, United States) to test their functionality and ease of use. The final bioreactor components were manufactured by computer-controlled milling and drilling. Cytocompatible and autoclavable materials were selected for all components in contact with medium or constructs.

### Bioreactor Testing

Preliminary tests on the whole bioreactor platform were performed for assessing ease of assembling, watertightness, functionality, and reliability. The 3D-printed prototypes of the sample holders were tested in-house in terms of coupling with the culture chamber components, and their holding performance was verified using silicone samples. Concerning the monitoring unit, calibration protocols were defined for both the sensors and the load cell, which was then characterized (see details in Supplementary Material). Ease of use, functionality, and reliability of the bioreactor were preliminarily tested in a cell culture laboratory. Laboratory operators assembled the bioreactor under hood, filled it with culture medium, placed it in incubator, and ran explanatory cyclic stimulation tests (10% strain, 1 Hz frequency) without constructs for 1–2 weeks.

### ECT Preparation and Culture

Annular silicone molds (external diameter = 16 mm, internal diameter = 10 mm, height = 3 mm, with a volume of approximately 400 μL) were manufactured for ECT casting. In each mold, a solution of 10 μL of thrombin (2.5 U/mL final, or 0.1 U/mL thrombin solution per mg fibrinogen, Sigma-Aldrich, United States) (Hansen et al., 2010; Morgan and Black, 2014), 23 μL of aprotinin (3000 KIU/mL, Sigma-Aldrich, United States) (Centola et al., 2013), and 116 μL of CaCl_2_ (40 mM, Sigma-Aldrich, United States) (Cholewinski et al., 2009) was injected. In parallel, freshly isolated neonatal rat cardiac cells (circa 80% CMs and 20% FBs) were isolated as previously described from 2–3-day-old Sprague Dawley rat hearts (Radisic et al., 2008), according to the Swiss Federal guidelines for animal welfare, and all procedures were approved by the Veterinary Office of the Canton Basel (Basel, Switzerland). Cardiac cells were suspended at a density of 18 × 10^6^ cells/mL (Vunjak-Novakovic et al., 2010) in 150 μL of high-glucose (4.5 g/L) Dulbecco’s Modified Eagle Medium (DMEM, Gibco, United States) supplemented with 1% fetal bovine serum (FBS, HyClone, United States), 1% penicillin/streptomycin (Sigma-Aldrich, United States), 1% L-glutamine (Sigma-Aldrich, United States), and 1% HEPES (Sigma-Aldrich, United States) and mixed with 100 μL of fibrinogen (25 mg/mL, Sigma-Aldrich, United States). Fibrin hydrogels were obtained by pouring the cell-fibrinogen solution within each mold already containing the thrombin solution. After 45 min in incubator at 37°C for complete hydrogel polymerization, the annular ECTs were removed from the molds, placed into a 6-well plate with 4-mL/well culture medium, and pre-cultured in incubator at 37°C and 5% CO_2_ for 5 days under static conditions (Zimmermann et al., 2000). Thereafter, ECTs were transferred into the bioreactor culture chamber and subjected to unidirectional cyclic stretch (sinusoidal waveform, 10% strain, 1 Hz) for an additional 4 days. As control experiment, ECTs were cultured statically for the entire duration of 9 days (Figure 1E). The culture medium, changed twice a week, consisted of low-glucose (1 g/L) DMEM, supplemented with 10% FBS, 1% penicillin/streptomycin, 1% L-glutamine, and 1% HEPES. At day 3, tranexamic acid (tAMCA, Sigma-Aldrich, United States) at a concentration of 160 g/mL was added to each well to delay fibrin hydrogel degradation (Cholewinski et al., 2009).

### ECT Assessments

#### Histology and Immunofluorescence Staining and CM Orientation Analysis

Cell distribution and tissue development and organization were evaluated by hematoxylin and eosin (H&E, Biosystems, Switzerland) staining on sample slices. Each construct was cut into two halves, and each part was fixed for 1 h in 1.5% paraformaldehyde (Sigma-Aldrich, United States), followed by an overnight treatment with 30% sucrose solution (Sigma-Aldrich, United States). Only the straight parts of the constructs (aligned to the stretch) were embedded in optimal cutting temperature compound (OCT, CellPath, United Kingdom) and successively cut in the longitudinal (parallel to the stretch) direction. Sections of 12 μm in thickness were cut in the cryostat by a microtome (Microm International GmbH, Germany) and stained with H&E according to standard protocols. Images were acquired with the Olympus BX63 microscope (Olympus, Japan). The most relevant cardiac markers, such as sarcomeric α-actinin, cardiac troponin I (cTnI), and connexin 43 (Cx43), were investigated by immunofluorescence analysis. The presence of cardiac FBs and cell proliferation were assessed by using anti-Vimentin and anti-ki67 antibodies, respectively. In detail, sections were incubated for 1 h in 0.3% Triton X-100 (Sigma-Aldrich, United States) and 2% normal goat/donkey serum (Sigma-Aldrich, United States) in PBS and successively for 1 h with the following primary antibodies: mouse monoclonal anti-sarcomeric α-actinin (Abcam, United Kingdom) at 1:400; mouse monoclonal anti-cardiac troponin I (Abcam, United Kingdom) at 1:100; rabbit polyclonal anti-Connexin 43 (Millipore, United States) at 1:100; goat polyclonal anti-Vimentin (Abcam, United Kingdom) at 1:100; and rabbit polyclonal anti-Ki67 (Abcam, United Kingdom) at 1:100. Sections were then incubated in the dark for 1 h with fluorescently labeled Alexa 488 and Alexa 647 anti-mouse and anti-rabbit antibodies (Invitrogen, United States) or Alexa 488, Alexa 546, and Alexa 647 anti-mouse, anti-rabbit, and anti-goat secondary antibodies (Invitrogen, United States) at 1:200. Nuclei were stained using 4′,6-diamidino-2-phenylindole (DAPI, Invitrogen, United States) at 1:50 for 1 h. Antibodies were diluted in 0.3% Triton X-100 and 2% normal goat/donkey serum in PBS. Images were taken by using an LSM-710 confocal microscope (ZEISS, Switzerland). The immunofluorescence staining images of statically (*n* = 3 biological samples) and dynamically (*n* = 4 biological samples) cultured ECTs collected at day 9 were then analyzed for quantifying the CM orientation. In detail, for each experimental group at least 5 immunofluorescence staining images of 3–5 constructs (from at least two independent experiments) were acquired using a 20X objective lens with the Olympus BX63 microscope (Olympus, Japan) and were analyzed using ImageJ software (NIH, United States). Cardiomyocyte orientation was quantified by calculating the angle between the primary axis of each troponin-positive cell and the *x*-axis of the image, corresponding to the direction of stimulation and considering the CMs aligned when the angle ranged angle ranged from 0° to 45° or from 135° to 180°. A two-way ANOVA statistical test was performed (*p* < 0.05). All data were represented as mean ± standard deviation.

#### DNA and Collagen Quantifications

To assess the amount of cells, DNA was quantified by CyQUANT^®^ Cell Proliferation Assay (Invitrogen, United States). Statically cultured constructs and straight parts of the dynamically cultured constructs were weighed and digested overnight at 57°C in 500 μL of proteinase K solution (1 mg/mL proteinase K, 50 mM TRIS, 1 mM EDTA, 1 mM iodoacetamide, and 10 mg/mL pepstatin-A; Sigma-Aldrich, United States) in double-distilled water or potassium phosphate buffer (Sadr et al., 2012). Working solutions were prepared according to the manufacturer’s protocols. Fluorescence was measured by a SpectraMax Gemini XS Microplate Spectrofluorometer (Molecular Devices, United States), with excitation and emission wavelengths equal to 485 and 583 nm, respectively. Each sample was measured in triplicate. The quantification of the ECT collagen content was carried out by hydroxyproline assay. After digestion in proteinase K (the same used for DNA quantification), samples were mixed with 12 M HCl, hydrolyzed at 120°C for 24 h, and then dried (Cigan et al., 2013). Subsequently, samples were mixed with 50 μL buffered chloramine T reagent (63.5 mg of chloramine T dissolved in 1 mL water, and then diluted with 1.5 mL *n*-propanol and 2.5 mL acetate–citrate buffer (pH 6); made fresh daily) and incubated for 20 min at room temperature. Further, 50 μL of dimethylaminobenzaldehyde (DMAB) solution mixed with perchloric acid were added (0.45 DMAB in 2 mL *n*-propanol and 1 mL perchloric acid 20%). The mass ratio of hydroxyproline to collagen was assumed equal to 1:10. The mixture was heated at 60°C for 20 min and cooled, and the absorbance determined at 550 nm. The hydroxyproline concentration was determined from a standard curve (stock solution of hydroxyproline: 1 mg/mL) (Hofman et al., 2011). The total amount of collagen was also normalized by the corresponding wet weight (ww) of the sample.

#### Mechanical Characterization

During the cyclic stretch culture, the bioreactor load cell was used at day 5 (start of the bioreactor culture) and day 9 (end of the bioreactor culture) for *in situ* measurement of the ECT mechanical response. The constructs were then collected from the bioreactor and hooked within a standard tensile testing machine (MicroBionix system, MTS Systems Corporation, United States), using a 2-N load cell. For both the bioreactor and the testing machine force measurements, (1) ECTs were pre-tensioned at an initial load of 0.02 N; (2) using a digital caliper, the initial length *L*_0_ between the two hooks was measured and the cross-sectional areas *A*_1_ and *A*_2_ of the two straight parts of the constructs were estimated; and (3) a series of 10 cycles (0–10% strain based on *L*_0_, 1 Hz) was performed. Force measurements were converted in engineering stress σ, defined as

$$\sigma =\frac{F}{\left(A_{1}+A_{2}\right)}$$

where *F* is the recorded force.

Stress *vs.* time curves generated from bioreactor and testing machine measurements at days 5 and 9 were compared in terms of peak-to-peak stress amplitude. The stiffness of the constructs, measured in terms of linear elastic modulus (E), was calculated from the testing machine data as the slope of the engineering stress–strain curves at increasing physiologic levels of strain (from 0 up to 30%). Moreover, imposing a ramp rate of 5 mm/min, ECTs were stretched until failure to assess the ultimate tensile strength (UTS, i.e., the maximum stress that the constructs can withstand before failure).

#### Electrical Functionality

ECT spontaneous beating and contractile activity upon external electrical pacing were monitored at days 5 and 9. A bright-field microscope IX81 (Olympus, Japan) at 2X/4X magnification was used, and images of specimen were captured. In detail, to evaluate the response to external pacing under controlled temperature of 37°C, each construct was placed within a Petri dish, filled with 5 mL of medium, in between two carbon rod electrodes connected to a custom-built electrical stimulator (Tandon et al., 2009). Electrical pacing (rectangular pulses, 2-ms duration) was applied at a rate of 1 Hz starting with a 1-V/cm electric field amplitude. The excitation threshold (ET), defined as the minimum electric field amplitude at which constructs are observed to beat synchronously, and the maximum capture rate (MCR), defined as the maximum pacing frequency for synchronous contraction using an electric field amplitude of two times the ET, were determined (Radisic et al., 2004). With the adopted microscope magnification levels, it was not possible to record the image of the entire construct while pacing. To overcome this limitation, different randomly sampled regions of the constructs (from 3 to 4, depending on the specimen) were image-recorded and analyzed. Once the image acquisition system was focused on the region of interest, the electric field amplitude was increased by single steps of 0.5 V/cm until the ET was reached. The MCR was determined imposing the electric field amplitude set at two times the ET and increasing the stimulation frequency by steps of 0.1 Hz, until the paced contractions became irregular or desisted at all. To assess the regularity and the frequency of contraction under external pacing, a video analysis of the constructs was performed using a customized image-processing algorithm. Each stimulation sequence was image-recorded [frames per second (fps) set in the range 20–40], and image frames were analyzed with ImageJ 1.47 software (NIH, United States). To assess the capability of the ECTs to follow synchronously the imposed pacing, the center of mass of the image-recorded construct portion was identified and its displacement with respect to its position in a reference frame was calculated and tracked (see details in Supplementary Material).

### Statistical Analysis

Statistical analysis was performed with GraphPad Prism software (GraphPad Software, Inc., United States). All data are presented as mean ± standard deviation. The one-way ANOVA multiple-comparison test was used to compare two different groups. In the figures, statistical significance is denoted as ^∗^ for *p*-value ≤ 0.05, ^∗∗^ for *p*-value ≤ 0.01, ^∗∗∗^ for *p*-value ≤ 0.001, and ^****^ for *p*-value ≤ 0.0001.

## Results

### Bioreactor Testing

Preliminary tests confirmed the bioreactor platform performance in terms of ease of assembling, watertightness, functionality, and reliability. In detail, the coupling and assembling/disassembling procedures of the mechanical components did not present any critical warnings, and the correct alignment of the culture unit, stimulation unit, and monitoring unit components was verified. Once assembled, the structural and electrical functionality of the bioreactor units was tested using the control unit user interface. In particular, the stimulation unit was tested in terms of accuracy and reliability, showing regular frequency and small oscillations in peak amplitude with no detectable drift from the imposed working parameters, while characterization tests confirmed the functionality of the load cell (see details in Supplementary Material). Thereafter, the bioreactor platform was preliminarily tested in a cell culture laboratory. The assembling procedure under laminar flow hood was easy and fast. Explanatory cyclic stimulation tests run for 1–2 weeks in incubator confirmed watertightness and sterility maintenance of the culture chamber.

### ECT Assessments

#### Remodeling, Histology, and Collagen/DNA Content

**FIGURE 2:**
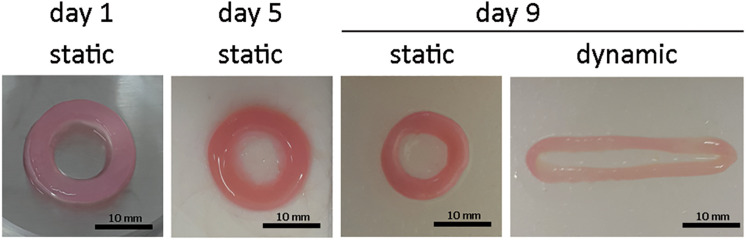
ECT shape remodeling. Representative macroscopic pictures showing the top view of an explanatory ECT at the beginning (day 1) and at day 5 of the culture, and following either static or dynamic culture (day 9). Scale bar = 10 mm.

**FIGURE 3:**
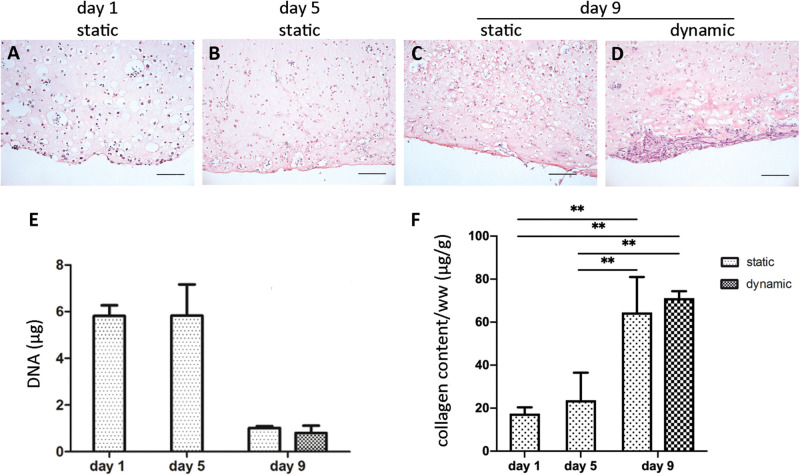
ECT cell distribution and DNA and collagen content. Representative H&E staining showing cell distribution within an explanatory ECT at the beginning (day 1) of the culture **(A)**, at day 5 **(B)**, and following either static **(C)** or dynamic **(D)** culture at day 9. Scale bar = 100 μm. Graphs showing the DNA amount **(E)** and the collagen content normalized by ww **(F)** for ECTs at the beginning (day 1) of the culture, at day 5, and following either static or dynamic culture (day 9). For all groups *n* = 3, ^∗∗^*p* ≤ 0.01.

The initial size of the ECTs was reproducible, but ECT shrinking was observed along the culture. In particular, the cross section of the constructs changed from an initial square shape with an estimated area equal to 5.2 ± 1.1 mm^2^ at day 1 to a circular shape with an estimated area equal to 4.0 ± 0.4 mm^2^ at day 5, and equal to 1.5 ± 0.7 mm^2^ and 0.9 ± 0.2 mm^2^ for static and dynamic culture at day 9, respectively (Figure 2). Representative images of the constructs stained for H&E at day 1 revealed that cells were homogeneously distributed within ECTs (Figure 3A). Concerning cell morphology, cells encapsulated within the fibrin hydrogel appeared round-shaped at day 1, and after 5 days of static pre-culture no appreciable differences in cell distribution and shape were observed (Figure 3B). At day 9, ECTs cultured under static conditions presented mostly round-shaped cells, sparsely distributed (Figure 3C). Differently, an increased cell density at the borders (at 50–200 μm from the edge) was observed in dynamically cultured ECTs, with the majority of the cells presenting an elongated morphology aligned with the construct edge, corresponding to the imposed stretch direction (Figure 3D). The amount of DNA was similar in the ECTs after 1 and 5 days; however, following 4 additional days, either in static or dynamic conditions the amount of DNA was reduced (Figure 3E). Construct remodeling and reorganization was investigated also by quantifying collagen deposition (Supplementary Figure 3) and collagen content per wet weight (Figure 3F). During static pre-culture (from day 1 to day 5), collagen content per ww did not significantly vary, while at day 9 it significantly increased (Figure 3F). This variation of the collagen content per ww along culture time is consistent with the observed remodeling of the constructs (Figure 2).

#### Cardiac Marker Immunofluorescence

**FIGURE 4:**
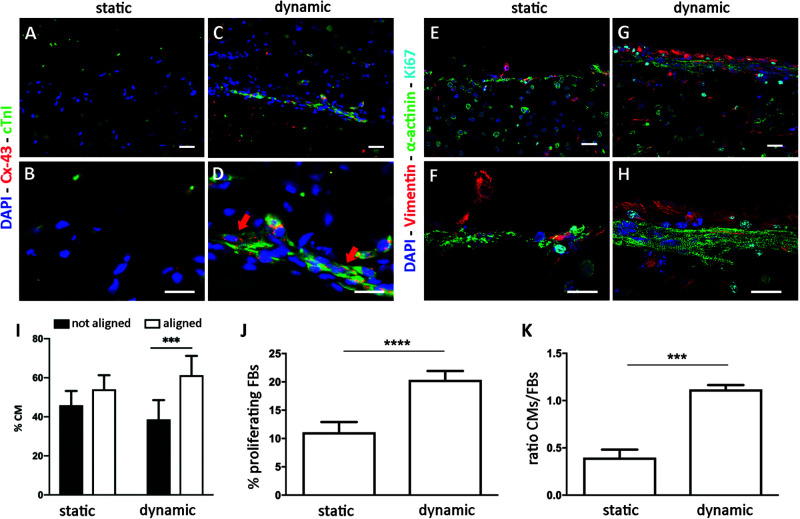
ECT biological characterization. Representative immunofluorescence images of statically **(A,B,E,F)** and dynamically **(C,D,G,H)** cultured ECTs at low (**A,C,E,G**, scale bar = 30 μm) and high (**B**,**D**,**F**,**H**, scale bar = 10 μm) magnification, stained for cardiac markers connexin-43 (Cx-43, red), Troponin I (cTnI, green), and α-sarcomeric actinin (α-actinin, green); for FB marker Vimentin (red); and for proliferating marker Ki67 (cyan). Nuclei are stained with DAPI (blue). **(I)** Graph showing the percentage of CMs aligned and not aligned with the direction of the mechanical stimulation, for statically (*n* = 3) and dynamically (*n* = 4) cultured ECTs. **(J)** Graph showing the percentage of proliferating FBs in statically and dynamically cultured ECTs at day 9. **(K)** Graph showing the ratio of CMs and FBs in statically and dynamically cultured ECTs at day 9. For all groups, *n* = 3, ^∗∗∗^*p* ≤ 0.001; ^****^p ≤ 0.0001.

Cardiomyocyte organization and maturation within the ECTs were assessed quantifying the cardiac marker α-sarcomeric actinin and the mature cardiac markers cTnI and Cx43. In particular, ECTs cultured in static conditions presented isolated and non-interconnected CMs both at day 5 (Supplementary Figure 4) and at day 9, with few cTnI-positive structures and absence of Cx43 gap junctions at day 9 (Figures 4A,B). In dynamically cultured ECTs, at day 9 most of the CMs close to the edges presented elongated and organized sarcomere units, positive for cTnI, typical of mature differentiation stage and Cx43 gap-junctional proteins (Figures 4C,D). In addition, the presence of striated α-sarcomeric actinin-positive structures was observed within ECTs cultured in dynamic conditions, where elongated cells exhibited the typical features of mature cardiac contractile units (Figures 4G,H). Such features were not detected in statically cultured ECTs, characterized by round-shaped cell morphology (Figures 4E,F). As concerns CM orientation, Figure 4I shows that in stretched ECTs almost 60% of CMs were aligned with the mechanical stimulation direction, while in statically cultured ECTs no significant differences were found. Cardiac FBs positive for DAPI and Vimentin, and negative for α-actinin, were observed in ECTs cultured under both dynamic and static conditions (Figures 4E–H). Notably, stretch stimulation promoted a significantly higher percentage of proliferating cardiac FBs (20% *vs.* 10% for dynamic culture and static control, respectively—Figure 4J), corresponding to Vim^+^ and Ki67^+^ cells. Furthermore, dynamic culture sustained the maintenance of the CM subpopulation, which resulted to be 1.2 times more than cardiac FBs, whereas in statically cultured ECTs cardiac FBs were two times higher in number than CMs (Figure 4K).

#### Mechanical Characterization

**FIGURE 5:**
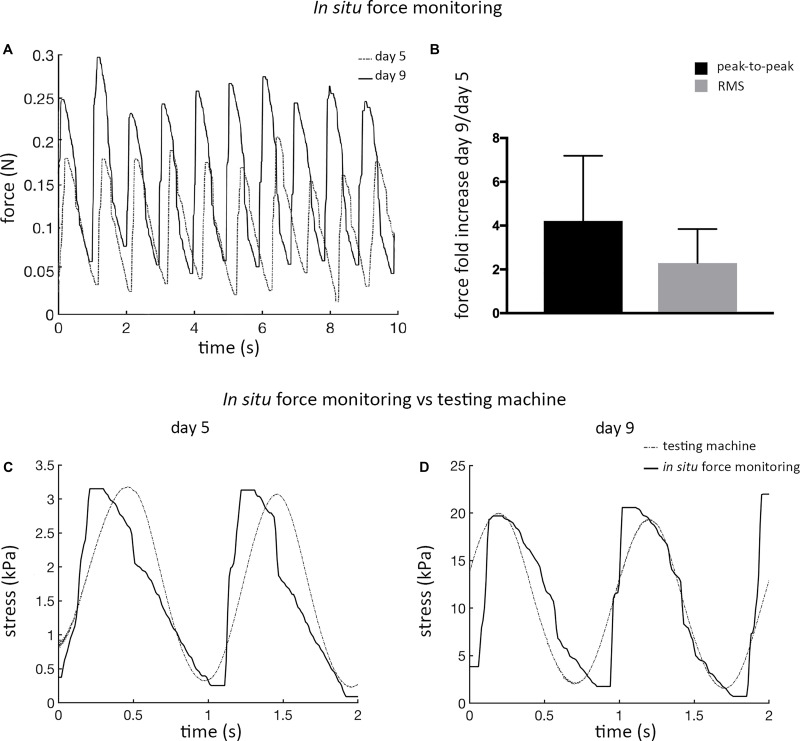
ECT *in situ* bioreactor force monitoring. **(A)** Real-time force curves measured *in situ* by the bioreactor load cell at day 5 and at day 9 for an explanatory ECT undergoing cyclic stretch. **(B)** Graph showing the increase in the peak-to-peak force amplitude and in the root mean square (RMS) force magnitude at day 9 in comparison to day 5, respectively (*n* = 3). Comparison of the stress *vs.* time curves obtained at days 5 **(C)** and 9 **(D)** from the bioreactor load cell (solid line) and from the standard testing machine (dotted line) data.

The mechanical response of ECTs undergoing cyclic stretch within the bioreactor was *in situ* measured at day 5 and at day 9, showing marked differences (Figure 5A). In detail, at day 9 ECTs exhibited a 2-fold increase in the root mean square (RMS) force magnitude and approximately a 4-fold increase in the peak-to-peak force amplitude in comparison with day 5 (*n* = 3, Figure 5B). For the sake of completeness, Supplementary Figure 5 shows an example of ECT passive forces measured *in situ* by the bioreactor load cell over 5 days of dynamic culture in a preliminary experiment. In accordance with data shown in Figure 5A, the magnitude of the measured forces increased along the culture (Supplementary Figure 5). As a check of consistency, stress *vs.* time curves obtained at days 5 and 9 from the bioreactor *in situ* monitoring and from the standard testing machine revealed comparable peak-to-peak stress amplitudes, thus confirming the reliability of the *in situ* monitoring setup (Figures 5C,D).

**FIGURE 6:**
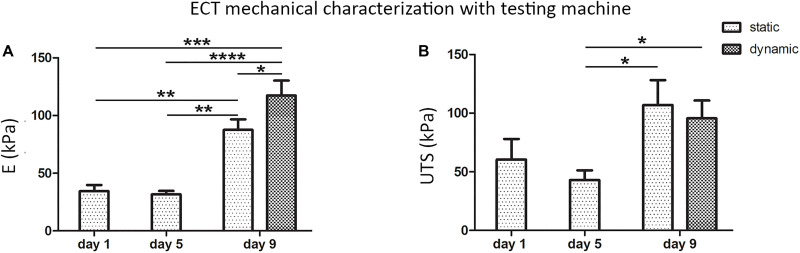
ECT mechanical characterization. Linear elastic modulus (E) values **(A)** and ultimate tensile strength (UTS) values **(B)** of freshly casted ECTs (day 1), ECTs at day 5, and statically and dynamically cultured ECTs at day 9 collected immediately after *in situ* force monitoring and measured using a standard testing machine. For all groups, *n* ≥ 3, ^∗^*p* ≤ 0.05; ^∗∗^*p* ≤ 0.01; ^∗∗∗^*p* ≤ 0.001; ^****^p ≤ 0.0001.

Freshly casted ECTs, statically cultured ECTs, and dynamically cultured ECTs collected immediately after bioreactor force monitoring were then mechanically characterized by using the standard testing machine (Figures 6A,B): (1) freshly casted ECTs and statically pre-cultured ECTs at day 5 presented a similar mechanical behavior in terms of linear elastic modulus (*E* = 34.4 ± 5.4 kPa and *E* = 31.7 ± 3.1 kPa, respectively), with no significant difference in terms of ultimate tensile strength (UTS = 60.5 ± 17.6 kPa and UTS = 42.9 ± 8.4 kPa, respectively), while (2) a statistically significant increase of the linear elastic modulus and ultimate tensile strength was observed at day 9 for ECTs cultured in both static (*E* = 87.5 ± 9.2 kPa, UTS = 107.0 ± 21.3 kPa) and dynamic (*E* = 117.3 ± 13.0 kPa, UTS = 95.6 ± 15.3 kPa) conditions. In particular, dynamically cultured ECTs exhibited an almost 4-fold increase of the linear elastic modulus compared with statically pre-cultured ECTs at day 5 and a 1.3-fold increase with respect to statically cultured ECTs at day 9 (Figure 6A).

#### Electrical Functionality

**FIGURE 7:**
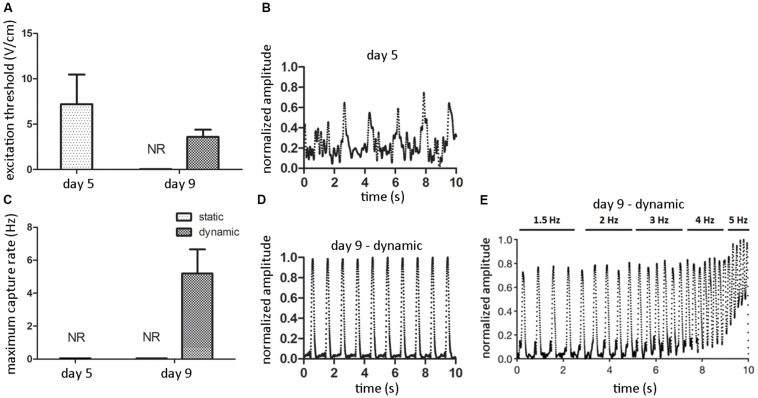
ECT electrical functionality. Graphs showing the excitation threshold (ET, **A**) and the maximum capture rate (MCR, **C**) of ECTs at day 5 and following static and dynamic culture at day 9 (NR = not responding to external electrical pacing, *n* = 3). Explanatory reconstructions from video-recorded images of the contraction amplitude waveforms (normalized to the maximum peak value) of ECTs exposed to electrical pacing at day 5 **(B)** and following dynamic culture at day 9 **(D)**. **(E)** Explanatory reconstruction of the contraction amplitude waveform of day 9-dynamic cultured ECT exposed to electrical pacing at increasing frequency.

After 5 days of static pre-culture, ECTs did not spontaneously beat. When electrically paced starting with 1 V/cm electric field amplitude (rectangular pulses, 2 ms duration, 1 Hz), they reached the excitation threshold at 7.2 ± 3.3 V/cm (Figure 7A), even though from the analysis of contractility an irregular, not synchronous beating over time emerged (Figure 7B). Although the pacing frequency was increased, it was not possible to determine the MCR of statically pre-cultured ECTs (Figure 7C). At day 9, any spontaneous beating was observed neither in dynamically cultured ECTs nor in static controls. Exposed to electrical pacing at increasing electric field amplitude (rectangular pulses, 2 ms duration, 1 Hz), the control group did not respond to the stimulation, while dynamically cultured ECTs reached the ET at 3.6 ± 0.8 V/cm (Figure 7A), with a regular, synchronous contractile activity over time (Figure 7D and Supplementary Video 1). Furthermore, imposing an electric field value 2-fold the ET and increasing the pacing frequency, dynamically cultured ECTs exhibited a MCR equal to 5.2 ± 1.46 Hz (Figure 7C). Finally, the contractile response of dynamically cultured ECTs exposed to increasing pacing frequencies (1.5–5 Hz) is presented in Figure 7E, where the capability of cyclically stretched ECTs to tune the beating rate accordingly to the imposed pacing frequency variation can be appreciated.

## Discussion

Biomechanics is a key player in governing and regulating myocardium development, homeostasis, and pathophysiology (Duscher et al., 2014; Voorhees and Han, 2015). During embryo development, CM contraction and blood flow exist within the primitive heart tube and influence the chamber curvature formation (Auman et al., 2007). Throughout neonatal development, myocardial stiffness increases together with an increase in heart contractility (Hopkins et al., 1973; Rodriguez et al., 2011). In cardiac disease development, alterations in cardiac function can be due to long-term changes in loading conditions, and in turn alterations in functionality and response of CMs and FBs can alter the mechanical properties of the heart with consequent maladaptive cardiac remodeling (McCain and Parker, 2011; Duscher et al., 2014; van Putten et al., 2016; Pesce and Santoro, 2017; Saucerman et al., 2019). As recently thoroughly investigated, cardiac cells are extremely sensitive to mechanical stimuli and react by activating defined mechanotransduction pathways, i.e., converting mechanical forces into biochemical signals that can lead to phenotypic changes (Lammerding et al., 2004; Sheehy et al., 2012; Lyon et al., 2015; Chimenti et al., 2017). For this reason, mechanical cues are paramount *in vivo* for the development of structurally and physiologically mature CMs and play a continuous, crucial regulatory role in cardiac growth and maintenance (Zhu et al., 2014). Moreover, *in vivo* physical forces are tightly regulated, or deregulated, not only in space but also in time (Valon et al., 2017). Thus, to promote *in vitro* a physiological-like maturation of cardiac tissue models, the provision of biomimetic physical conditioning with tunable intensity over time (Ronaldson-Bouchard et al., 2019), properly adapted to the actual maturation stage of the cultured constructs, is crucial.

Inspired by these arguments, we developed a bioreactor platform for biomimetic dynamic culture of cardiac tissue models and *in situ* non-destructive monitoring of their mechanical response. The rationale behind the design of the proposed bioreactor platform was to develop a unique, reliable, and versatile culture device able (1) to deliver tunable native-like or pathological-like cyclic stretch for the maturation of 3D cardiac tissue models or the development of diseased cardiac tissue models and (2) to *in situ* measure their passive mechanical response along the culture in a non-destructive manner, in the future perspective of continuously adapting the load to the actual maturation stage of the cultured constructs. In terms of mechanical stimulation, the adopted engineering and technological solutions led to the implementation of a displacement-controlled strategy allowing a wide range of well-defined physiological/pathological-like stretching and frequencies. In particular, the use of a linear voice coil motor enabled high displacement amplitude and frequency. Moreover, the adopted feedback-loop control strategy guaranteed high-fidelity tracking of the motor reference position with consequent high-displacement resolution even in case of variations of the environmental conditions (e.g., temperature, humidity, and pressure), which could induce unexpected friction. Further preliminary tests, carried out in a cell culture laboratory running explanatory stimulation tests without constructs, proved the bioreactor watertightness and sterility maintenance and confirmed that the bioreactor is an easy-to-use platform.

Proof-of-concept experiments were then performed on specifically developed fibrin-based annular ECTs, tested under static and dynamic conditions. These experiments had the dual aim of (1) testing the bioreactor performance in terms of cyclic stretching and *in situ* monitoring of the mechanical response of cardiac tissue models and (2) investigating the effect of cyclic mechanical stimulation on cardiac tissue organization, maturation, mechanical properties, and electrical functionality. In detail, ECTs were prepared, pre-cultured for 5 days in static conditions, and successively transferred into the bioreactor culture chamber to be cyclically stretched (sinusoidal waveform, 10% strain at 1 Hz) for an additional 4 days. In parallel, corresponding static control experiments were carried out. At the end of the culture, dynamically cultured ECTs presented increased cell density and elongated cells at the borders, parallel to the stretch direction (Figure 3D). This was confirmed by immunofluorescence analyses, which revealed a network of cTnI/sarcomeric α-actinin-positive CMs longitudinally oriented along the longitudinal cross section of the constructs (Figure 4I) and prevalently localized on lateral edges, expressing Cx43 electrical gap junctional proteins, all typical features of mature cardiac contractile units (Figures 4C,D).

Although the total amount of DNA decreased in both culture conditions at day 9 compared to day 5 (Figure 3E) and FBs proliferated more in dynamic than in static culture (Figure 4J), the number of CMs was still superior to FBs following stretching (Figure 4K), most likely due to a better CM survival under dynamic conditions. On the contrary, statically cultured ECTs presented round-shaped, isolated, non-interconnected CMs, with low or even lacking expression of mature cardiac markers (Figures 4A,B). Under static culture, although FBs proliferated less (Figure 4J), the final FB number was two times higher than the CM number (Figure 4K), possibly linked to the apoptosis of several CMs following prolonged static conditions. Mechanical stimulation might indeed have promoted the further maturation of CMs and the exchange of nutrients, oxygen, and waste products, which in static conditions was limited to the diffusion process.

Concerning ECT maturation in terms of matrix remodeling/generation and mechanical properties, the total amount of collagen did not vary significantly along the culture (Supplementary Figure 3), although it has to be considered that this information refers to the total amount of proteins and it does not take into account the fiber types, morphology, and organization, while the collagen content per ww increased with the time in culture (Figure 3F), consistently with the construct remodeling (Figure 2). *In situ* monitoring allowed measuring the ECT passive force response to cyclic stretch, showing a force increase in terms of both RMS force magnitude and peak-to-peak amplitude along the culture (Figures 5A,B). Moreover, stretched constructs presented increased mechanical properties (E and UTS) over culture time (Figures 6A,B). Since the total collagen content remained constant, it can be argued that the observed differences in mechanical properties are related to the remodeling of the constructs.

Finally, ECTs were tested in terms of electrical functionality by investigating their regularity and frequency of contraction under external electrical pacing. ECTs did not spontaneously beat, neither at the end of the pre-static culture (day 5) nor at day 9. When electrically paced, only stretched ECTs presented a decreasing ET and an increasing MCR over time (Figures 7A,C), and at day 9 they regularly and synchronously beat following the external pacing (Figure 7D), while statically cultured constructs did not respond. This observation is further evidence of the instrumental role of cyclic stretch in promoting the functional coupling and the development of adult-like CMs in cultured ECTs, able to respond synchronously to increasing pacing frequency (Figure 7E). Although our preliminary biological results are not representative of cardiac tissue models exhibiting uniform and complete cardiac cell maturation, with this study we confirmed the developmental effect of cyclic stretch on ECT organization and maturation, in accordance with previous studies (Liaw and Zimmermann, 2016; Parsa et al., 2016; Stoppel et al., 2016). Time in culture under mechanical stimulation longer than 4 days should be able to provide a more uniform maturation and organization of CMs.

The reliability of the bioreactor platform was thus verified both for the engineering design and for the feasibility of dynamically culturing 3D ECTs. Moreover, the comparison of the stress *vs.* time curves obtained using the bioreactor load cell and the testing machine (Figures 5C,D) confirmed the reliability of the *in situ* monitoring setup for non-destructive measurement of passive mechanical response of the constructs to cyclic stretch.

In the past, a plethora of studies used dynamic culture devices developed to expose ECTs to cardiac-like isotonic load by using actuators (Fink et al., 2000; Zimmermann et al., 2002; Naito et al., 2006; Birla et al., 2007; Tulloch et al., 2011; Salazar et al., 2015). However, the mechanical response of the cultured ECTs was not monitored along the culture in any of these studies, where the constructs were mechanically characterized only at the end of the culture by using, e.g., testing machines or organ baths, thus providing only end-point measurements. On the other hand, in other studies flexible posts were adopted to stimulate ECTs by auxotonic load (Zimmermann et al., 2006; Hansen et al., 2010; Schaaf et al., 2011; Boudou et al., 2012; Mannhardt et al., 2016). This made possible the implementation of automated methods for the evaluation of the contractile activity of the constructs along the culture but required long-term culture periods and led to ECTs characterized by structural and functional properties typical of postnatal myocardium. To our knowledge, only Kensah et al. (2011) combined cyclic stretch and *in situ* monitoring of mechanical behavior within a bioreactor, using a device designed for dynamically culturing and real-time measuring the contraction force of spontaneously beating miniaturized ECTs, based on a matrix of collagen and Matrigel.

Differently, our bioreactor platform was designed to stretch and *in situ* measure along the culture the passive mechanical response of macroscopic constructs, which can be of different shape and composition. By comparing the force measurements acquired along the culture, related to the mechanical properties of the constructs as shown, our device allows to monitor the construct maturation trend without interrupting the culture. This enables reducing the number of time-point analyses and thus of replicate samples. Furthermore, this bioreactor platform could be exploited for continuously adapting the stimulation to the actual development of the constructs. In principle, by equipping the platform with an additional feedback-loop control between the *in situ* force monitoring and the motor, this approach would enable the biomimetic adaptation of the mechanical load to the developmental stage of the cultured ECTs. Moreover, the integration of a compact digital camera and an autofocus lens for macro photography located perpendicularly to the bioreactor optical access would allow acquiring real-time images of the constructs during dynamic culture. This feature could be exploited for automatically *in situ* measuring the ECT geometry, ultimately providing real-time mechanical characterization of the constructs in terms of stress–strain curves.

In terms of versatility, in the future the proposed platform will be equipped with an electrical stimulation unit to provide individual, alternate, and/or combined mechanical and electrical stimulations to mimic the complex biophysical cardiac environment present *in vivo*. Furthermore, enabling a wide range of displacements and stimulation frequencies, this bioreactor could be used for several applications in different research fields, such as skeletal muscle or tendon/ligament tissue engineering or for disease modeling imposing pathological physical conditioning. In conclusion, the proposed bioreactor platform represents a powerful tool for *in vitro* investigation and ultimately production of functional engineered constructs.

## Data Availability Statement

The raw data supporting the conclusions of this article will be made available by the authors, without undue reservation, to any qualified researcher.

## Ethics Statement

The animal study was reviewed and approved by the Veterinary Office of the Canton Basel (Basel, Switzerland), according to the Swiss Federal guidelines for animal welfare.

## Author Contributions

DM, GP, AM, and UM conceived the study. GP and DM conceived the bioreactor platform design. AR, GP, and RG developed the control unit and the control software. AT and RG selected the motor. GP, GI, DM, CB, AA, and UM conceived and designed the in-house experiments. GP and AM conceived the biological experiments. GP and GC performed the biological experiments and assessments. GP, GI, AP, and AM analyzed the biological data. DM and GP reviewed the state of the art. GI and DM prepared the figures. AM and UM contributed to the writing of the manuscript. DM, GI, and GP wrote the manuscript. All the authors contributed to the article and approved the submitted version.

## Conflict of Interest

The authors declare that the research was conducted in the absence of any commercial or financial relationships that could be construed as a potential conflict of interest.
