# Supplementary material for: Bioreactor Platform for Biomimetic Culture and in situ Monitoring of the Mechanical Response of in vitro Engineered Models of Cardiac Tissue
Source: Front Bioeng Biotechnol. 2020 Jul 14;8:733. doi: 10.3389/fbioe.2020.00733 (PMC7381147; doi:10.3389/fbioe.2020.00733)
Supplement: Supplementary file 1 [file Presentation_1.pdf]

Supplementary Material

1 Supplementary Figures and Videos

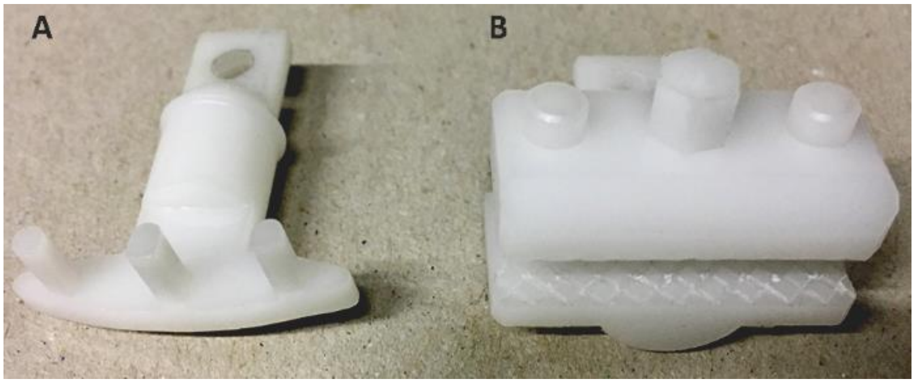

Supplementary Figure 1. Frontal view of the interchangeable sample holders, designed for ring-shaped constructs (**A**) or for patch-shaped constructs (**B**).

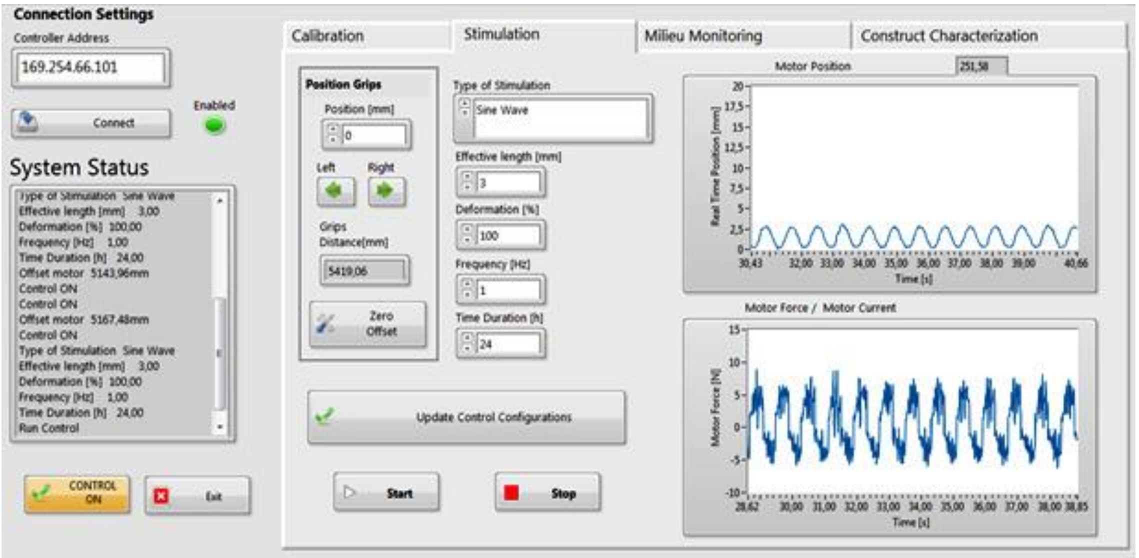

Supplementary Figure 2. Explanatory picture of the user-friendly software interface, equipped with the following panels: 1) Calibration, for guiding the calibration of the sensors and of the load cell; 2) Stimulation, for setting the mechanical stimulation parameters and monitoring the motor working conditions; 3) Milieu Monitoring, for monitoring/recording the environmental data collected by the sensors; 4) Construct Characterization, for monitoring/recording the passive mechanical response of the constructs collected by the load cell.

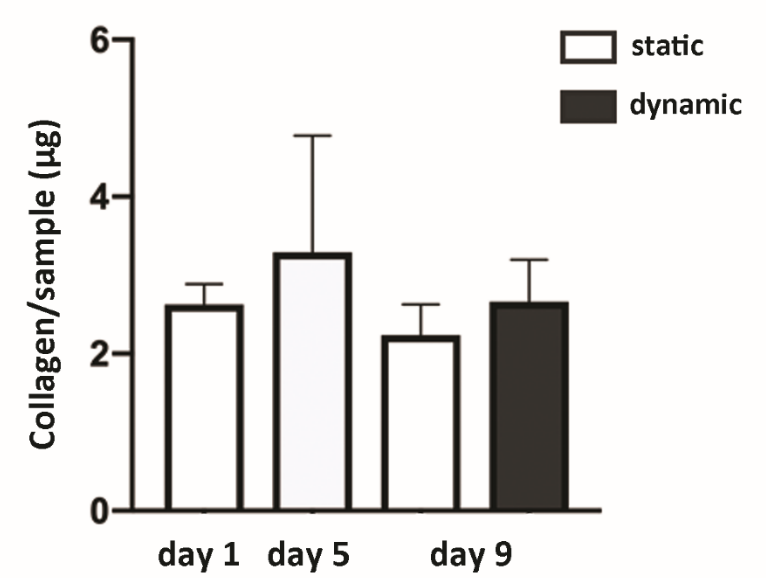

Supplementary Figure 3. Graph showing the total amount of collagen per sample at the beginning of the culture (day 1), at day 5, and following either static or dynamic culture (day 9), ($p>0.05$).

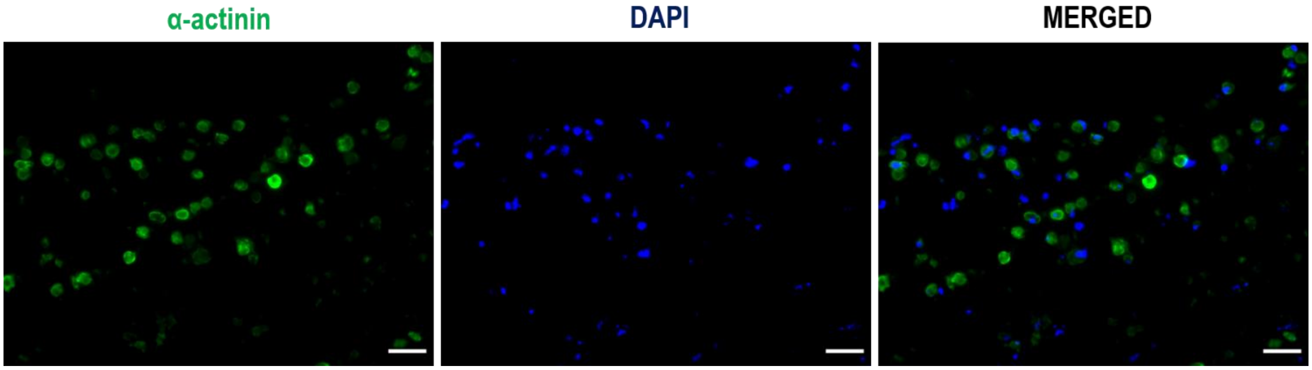

Supplementary Figure 4. Representative immunofluorescence images of statically cultured ECTs at day 5 stained for cardiac marker α-sarcomeric actinin (α-actinin, green) and nuclei marker DAPI (blue). Scale bar = 30 µm.

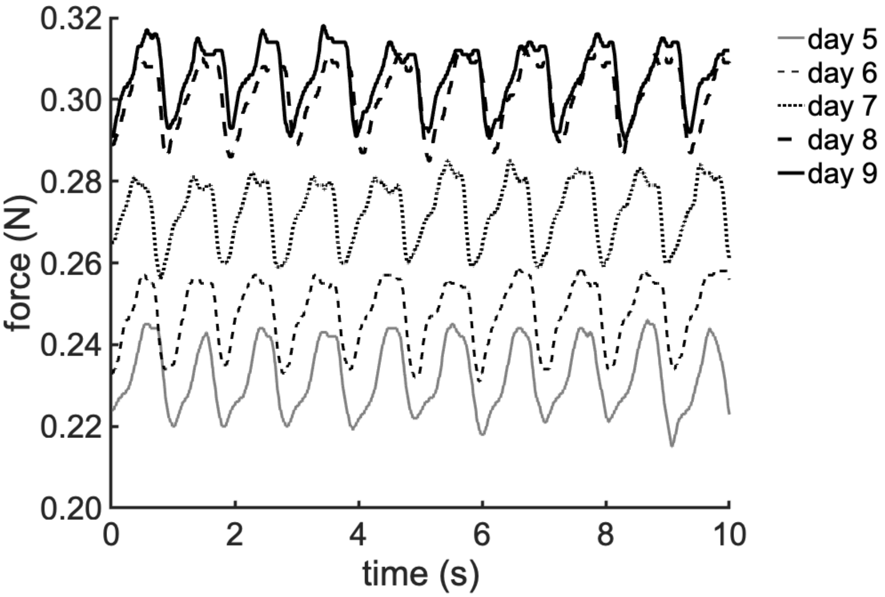

Supplementary Figure 5. Real-time passive force curves measured *in situ* by the bioreactor load cell during the culture of an explanatory ECT undergoing cyclic stretch.

Supplementary Video 1. Video of an explanatory dynamically cultured construct collected at day 9 and exposed to external electrical pacing (rectangular pulses, 2 ms duration, 1 Hz). File type WMV.

2 Supplementary Information

2.1 Structure and testing of the motor control

The displacement of the motor is controlled by a cascade control strategy, based on feedback loops among the motor, the linear position transducer and the control unit. In detail, the motor control structure consists of: 1) an inner current feedback loop, designed as a Proportional-Integral (PI) compensator and based on the shunt resistance inside the power module; 2) an outer displacement feedback loop, composed as a Proportional-Integral-Derivative (PID) compensator and based on a dedicated displacement transducer; 3) a feed-forward loop to compensate friction effects and to provide effective mitigation of the dry friction phenomena that can be due to the contact between moving parts (Supplementary Figure 6).

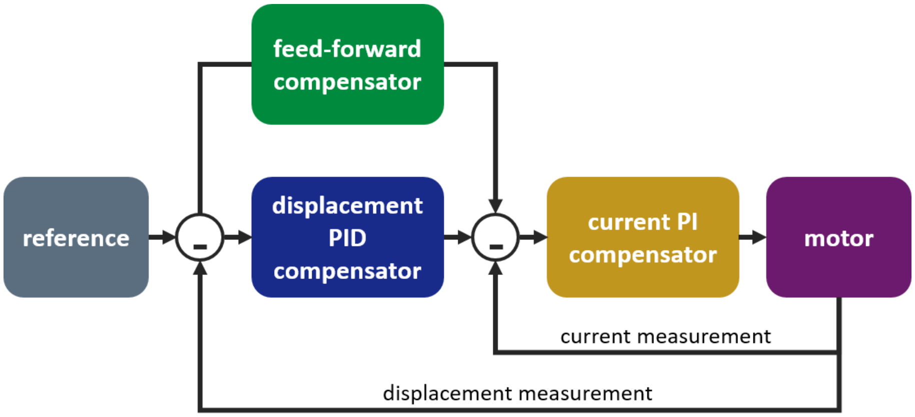

Supplementary Figure 6. Scheme of the motor control structure composed of: inner current feedback loop; outer displacement feedback loop; feed-forward friction loop.

For tuning the motor control loops and for defining the starting values for the PID compensator, a numerical lumped model of the motor control structure was developed in MATLAB (MATLAB&Simulink, MathWorks, USA). Lumped model parameters were set based on hardware features and on the required performance. The feed-forward current gain was identified by estimating the Coulomb friction force offset of the device. For simulation purposes, the model was executed using the ode23s variable-step integration method. Starting from the PID values obtained from the simulations, a wide range of motor working conditions, in terms of displacement (0.5 – 3.0 mm) and frequency (1 – 6 Hz), were simulated, and the best gain coefficients were obtained for a specific working range (0.5 – 3.0 mm, 1 Hz). The agreement between the real motor displacement and the modeled input reference, in terms of waveform amplitude and frequency, was evaluated (Supplementary Figure 7). For low displacement values (i.e., 0.5 – 1.0 mm), the motor displacement was regular in frequency even with moderate oscillations in the peak amplitudes (~ 20-13% discrepancy with respect to the reference values, respectively). For wider displacement values (i.e., 1.5 – 3.0 mm), the motor performance improved with increased repeatability, assuring the regular working frequency and small oscillations in peaks amplitude (~ 9-3% discrepancy with respect to the reference values, respectively). The observed, acceptable discrepancies between the reference waveforms and the real waveforms should be ascribed to hysteretic nonlinearities of the system, which were not included in the model.

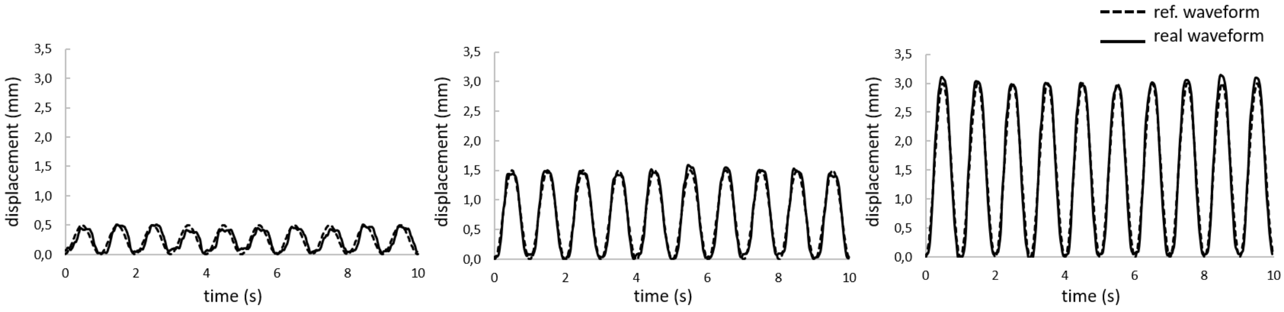

Supplementary Figure 7. Reference and real waveforms imposing a motor displacement of 0.5 mm, 1.5 mm, and 3.0 mm at 1 Hz, respectively.

2.2 Characterization of the load cell

In the adopted architectural set-up, the load cell (XFTC300, Measurement Specialties TE Connectivity, USA) is connected to the permanent holder through-shaft for *in situ* monitoring the passive mechanical response of the constructs along the culture. Although machining tolerances and the correct assembly should ideally guarantee coaxiality and absence of any torque, the presence of possible, small misalignments could induce friction and affect the sensing performance of the load cell. To assess possible sensing inaccuracy, a characterization of the load cell was performed using a calibrated instrumentation spring. The spring stiffness was firstly evaluated by using a standard tensile testing machine (Microbionix system, MTS Systems Corporation, USA) equipped with a 10N full scale range load cell. From the linear regression analysis of the recorded force-displacement data, the curve slope in the linear region was calculated (spring stiffness = 0.057 ± 5.19^{-5} N/mm, n = 3). Afterwards, the spring was housed within the culture chamber in dry conditions (Supplementary Figure 8) and a ramp displacement with a nominal speed of 5 mm/min and a total displacement of 3 mm was imposed. The spring stiffness measured within the bioreactor resulted to be 0.052 ± 9.54^{-4} N/mm. In the adopted architectural set-up, the stiffness value of the spring obtained using the bioreactor load cell differed no more than 10% with respect to the reference value, indicating that the load cell can provide an indicative estimation of the cultured construct stiffness.

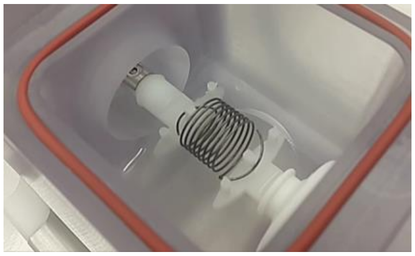

Supplementary Figure 8. Picture of the instrumentation spring housed with the bioreactor culture chamber for the load cell characterization tests.

2.3 Contractility video analysis

The contractility video analysis (in terms of regularity and frequency) of the cultured constructs exposed to external electrical pacing was performed using a customized image-processing algorithm. Technically, along each electrical stimulation sequence the constructs were image-recorded (frames per second (fps) set in the range 20 - 40), and the acquired image frames were analyzed with ImageJ 1.47 software (NIH, USA). In detail, each image-recorded frame of a construct portion (Supplementary Figure 9A) was scaled according to the magnification. A brightness threshold was set to operate quantitatively on the images, and a region of interest (ROI) comprising the construct portion and its surrounding space of movement were selected (Supplementary Figure 9B). For each image-recorded frame, the center of mass of the construct portion was identified and its displacement (d_i) with respect to its position in a reference frame, was calculated as:

$$d_i = \sqrt{(x_i - x_1)^2 - (y_i - y_1)^2} \quad \text{(Eq.1)}$$

where (x_1, y_1) and (x_i, y_i) are the center of mass coordinates in the reference frame and in the *i-th* frame, respectively. The calculated displacement along time was used to assess the capability of the constructs to follow synchronously the external imposed pacing.

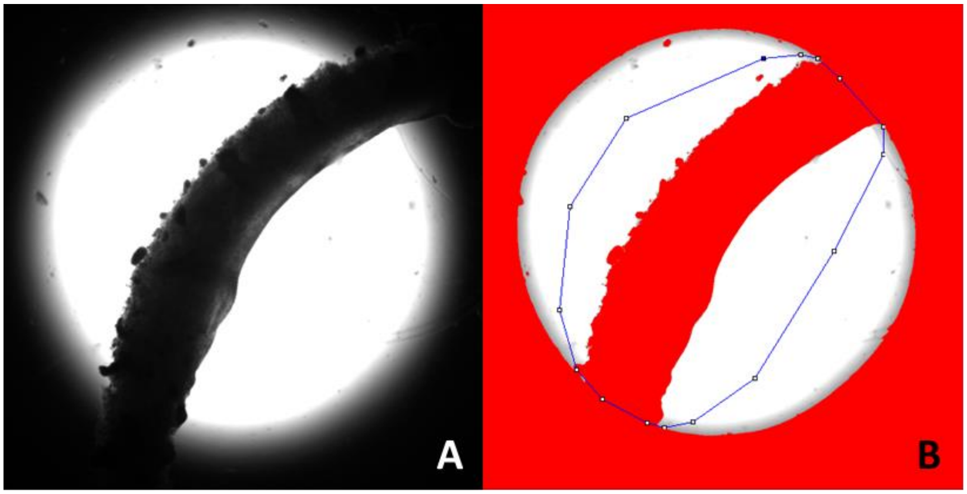

Supplementary Figure 9. Contractility video analysis. (**A**) Explanatory image-recorded frame of a construct portion during external electrical pacing. (**B**) Explanatory selection procedure of the ROI.
